# Assessing the impact of the COVID-19 pandemic on neglected tropical diseases in India: a perspective

**DOI:** 10.3389/fpubh.2024.1366484

**Published:** 2024-12-18

**Authors:** Ilham Zaidi, Jagadeeswari Vardha, Abdul Khayum, Sahifa Anjum, Shikhar Chaudhary, Aditi Bakshi, Jasmeen Kaur Gill

**Affiliations:** ^1^International Society for Chronic Illnesses, New Delhi, India; ^2^The Achutha Menon Centre for Health Science Studies, Sree Chitra Tirunal Institute for Medical Sciences and Technology, Trivandrum, India; ^3^Department of Population Health, University of Glasgow, Scotland, United Kingdom; ^4^Department of Respiratory Medicine, JSSMC, Mysuru, India; ^5^Dr. S.D. Gupta School of Public Health, IIHMR University, Jaipur, India; ^6^Indian Institute of Public Health Delhi, New Delhi, India

**Keywords:** neglected tropical diseases, corona virus disease 2019, COVID-19 pandemic, healthcare systems and management, low-and middle-income countries

## Abstract

The COVID-19 pandemic has significantly challenged healthcare systems worldwide, particularly in India, a country already burdened with a high prevalence of Neglected Tropical Diseases (NTDs). This perspective examines the pandemic’s direct and indirect impacts on the prevalence, diagnosis, and management of NTDs in India. Using a narrative review approach, we analyzed literature published between January 2020 and September 2023 from databases such as PubMed, Scopus, and Google Scholar, along with grey literature. The focus was on studies reporting the pandemic’s influence on NTDs, especially among vulnerable populations in both rural and urban settings. The review incorporated 49 studies, revealing a twofold impact of COVID-19 on India’s healthcare. Directly, the surge in COVID-19 cases strained healthcare resources, disrupted services, and overwhelmed healthcare personnel. Indirectly, the pandemic exacerbated the burden of NTDs by delaying diagnoses, limiting access to treatment, and redirecting resources toward pandemic response efforts. These findings highlight the urgent need for resilient healthcare strategies that address both the immediate and long-term impacts of the pandemic on NTDs. By understanding and mitigating these effects, policymakers and public health experts can better protect vulnerable populations from the compounded challenges posed by these neglected diseases.

## Introduction

The Neglected Tropical Diseases (NTDs) are a subset of infectious diseases that are prevalent in tropical and subtropical regions of the world affecting more than 1 billion people from Low-middle income countries (LMICS). They encompass a diverse range of infections caused by various pathogens, including parasites, bacteria, and viruses (1, 2). In the early 2000s, the World Health Organization initially identified and focused on 17 diseases in their portfolio, as research and attempts to treat NTDs progress, more diseases were added to the list in 2016, bringing the total to 20. Some of the most common NTDs include lymphatic filariasis, dengue fever, leishmaniasis, soil-transmitted helminthiasis, schistosomiasis, and onchocerciasis, among others (3). The term “neglected Tropical Diseases” highlights two key characteristics associated with the disease occurrence in the population:

1. Predominance in Poverty- Stricken areas: NTDs primarily predominate in the tropical and subtropical regions, disproportionately affecting populations living in remote rural communities, urban slums and migrant populations (4). Consequently, these diseases thrive in settings with limited availability of necessary resources like sanitation, safe drinking water, and healthcare facilities, leading to their widespread occurrence (3).
2. Historical Neglect: These diseases are referred to as “neglected” due to their historical status of receiving low priority in terms of research funding, drug development, and public health attention. The focus and resource allocation, especially during the Millennium Development Goals 6, this negligence is obvious. At that time, broader global goals were given higher importance, such as reducing child and maternal mortality and waging a fierce war on HIV/AIDS, malaria, and tuberculosis (1).

### Prevalence of neglected tropical diseases in India

India, with its diverse climate and geography, provides a fertile ground for the prevalence of various NTDs. According to the WHO, India is the country experiencing the world’s largest burden of at least 11 major NTDs in terms of the total number of cases (5).

Lymphatic filariasis (LF), caused by the parasitic worm Wuchereria bancrofti, is a major NTD in India. The WHO estimated that India accounted for approximately 40% of the global LF burden, which is prevalent in 256 districts with nearly 650 million people at risk of getting the disease (6).
Dengue fever, transmitted by the Aedes mosquito, is another prominent NTD and endemic in almost all states in India. The country experiences recurrent outbreaks, with millions of cases reported annually and in 2018, more than 1 lakh cases were identified with 172 deaths (7). The prevalence of dengue varies across different regions, with urban areas often witnessing higher transmission rates.
Soil-transmitted helminthiasis (STH), referring to intestinal helminthic infections caused by parasitic worms such as roundworms, hookworms, and whipworms. These infections are a significant public health concern and 21% of the global prevalence is seen in India (8). They tend to be most prevalent among rural communities residing in warm and humid regions with inadequate sanitation facilities, However, urban communities are facing increased number of STH cases in India (9).
Other NTDs like visceral leishmaniasis (kala-azar), schistosomiasis, and onchocerciasis have localized pockets of endemicity in specific regions of India. Efforts have been made at both national and international levels to combat these diseases. Various public health programs and initiatives have been implemented to control and eliminate NTDs in India (5).

Moreover, the influence of NTDs not only escalates owing to socio-economic and environmental factors, but the recent COVID-19 pandemic has also directly and indirectly affected the management of Neglected Tropical Diseases (NTDs). Although the COVID-19 pandemic has had an impact on every sector, the crisis is severe for many countries’ already overburdened health systems. India, being the country with high prevalence of NTDs was severely impacted during the pandemic period due to resource diversions, limited availability of health services for patients with NTDs, and impact on surveillance and data collection procedures. It is crucial to evaluate the affecting factors and implement necessary prevention measures to tackle the prevalence and incidence of NTDs during the pandemic period and implement the same for any future outbreaks. However, there is a gap in research studies reviewing the impact of COVID-19 pandemic on the healthcare services of NTDs. This research review aimed at comprehensively examining the impact of the COVID-19 pandemic on healthcare systems in India and assess its direct and indirect effects on the prevalence, diagnosis, and management of NTDs in India.

## Methodology

### Search methods

A narrative review approach was utilized, involving a comprehensive search of multiple electronic databases, including PubMed, Scopus, and Google Scholar. The search was designed with input from co-authors, adhering to accepted narrative review procedures. Relevant search terms and keywords related to COVID-19, neglected tropical diseases, and India were employed to capture pertinent material.

To ensure that appropriate content was retrieved, the search strategy was both extensive and focused, incorporating free-text terms and medical subject headings (MeSH). Filters were applied to limit the search results to research conducted in India and published in English. Grey literature sources, such as government publications, conference proceedings, and organization websites, were also reviewed to supplement the database search.

### Search methods

A systematic search of multiple electronic databases, including PubMed, Scopus, and Google Scholar, was conducted to identify relevant studies published between January 2020 and September 2023. The search plan was created with the help of co-authors and according to accepted scoping review procedures. To find relevant material, important search phrases and keywords pertaining to COVID-19, neglected tropical diseases, and India were utilized.

To ensure that appropriate material would be retrieved, the search strategy was created to be both thorough and targeted. It included both free-text terms and medical subject headings (MeSH). To restrict the search results to research done in India and published in English, filters were used. In order to augment the database, grey literature sources like government publications, conference proceedings, and organization websites were also searched.

### Study selection criteria

Studies which met the following requirements were included to the review:

Relevance: Research on how COVID-19 affects neglected tropical diseases in India in terms of prevalence, diagnosis, or treatment.

Study Design: Observational studies, surveys, case reports, and qualitative interviews were among the quantitative and qualitative research that were taken into consideration.

Population: Research on human populations in India impacted by neglected tropical diseases, encompassing all age ranges and socioeconomic backgrounds.

Inclusion criteria:

- Studies published between January 2020 and September 2023.
- Studies conducted in India.
- Articles focusing on the impact of COVID-19 on healthcare systems and NTDs.
- Both primary research studies (quantitative and qualitative) and systematic reviews.

Exclusion criteria:

- Studies not conducted in India.
- Studies not focusing on neglected tropical diseases.
- Studies lacking relevance to the impact of COVID-19.

The selection process involved screening titles and abstracts for relevance, followed by a full-text review of potentially eligible studies. Two independent reviewers conducted the screening process, with discrepancies resolved through consensus or consultation with a third reviewer if necessary. To minimize bias, a standardized data extraction form was used to extract relevant information from included studies, including study characteristics, key findings, and methodological details. The review incorporated 49 studies, including both quantitative and qualitative research designs.

### The COVID-19 pandemic

The novel coronavirus SARS-CoV-2, which emerged in late 2019, has spread to 192 countries, posing a significant global threat (10). The disease is characterized by rapid human-to-human transmission with a spectrum of clinical manifestations, ranging from mild respiratory symptoms to severe pneumonia, multi-organ failure, and death (11). In response to the escalating threat, governments and health authorities worldwide implemented a range of containment measures, including social distancing, lockdowns, and the deployment of vaccination campaigns.

### Impact on healthcare systems globally

The gradual spread of the virus across all settings throughout the early stages had a considerable impact on the delivery of health services. It presented difficulties for the administration of medical supplies, facility use, and human resources in the health sector (12).

Even countries with robust healthcare infrastructure and resources encountered significant hurdles during the pandemic. Shortages of critical supplies, including personal protective equipment (PPE), ventilators, and essential medical supplies, were a common issue across many well-resourced healthcare systems. The sudden and widespread demand for these items strained supply chains and highlighted the need for more resilient and responsive medical supply systems (13). Shortages of healthcare professionals and concerns about their safety became critical issues. Additionally, the pandemic emphasized the interconnectedness of global health and the importance of international cooperation. Information sharing and collaboration among countries and organizations became vital tools in responding effectively to the crisis (14).

Furthermore, the virus’s ability to mutate and the emergence of new variants raised concerns. These variants could potentially impact vaccine efficacy and prolong the timeline for achieving herd immunity. Continuous surveillance, research, and adaptation of vaccines to new variants became imperative in staying ahead of the virus’s evolving nature. The pandemic spurred remarkable progress in vaccine development, but the journey towards global herd immunity remained fraught with challenges (15).

### Impact on healthcare systems in India

The COVID-19 pandemic placed an unprecedented strain on healthcare systems by overtaking the functioning of other health facilities across the globe, including India (16). The outbreak paralyzed the health infrastructure mainly because of shortage in medical equipment and critical resources such as ventilators, oxygen, personal protective equipment (PPE), and skilled healthcare personnel (17). Here are some keyways in which the pandemic affected healthcare in India:

#### Overwhelmed healthcare system

The surge in COVID-19 cases overwhelmed the healthcare system, particularly in densely populated urban areas. Hospitals faced shortages of critical resources such as hospital beds, ventilators, oxygen supplies, and personal protective equipment (PPE) for healthcare workers (18).

#### Redirection of resources

With the priority on treating COVID-19 patients, resources that were originally allocated for non-COVID medical conditions were diverted. Elective surgeries and routine medical care were often postponed, leading to challenges for patients with chronic conditions or other urgent healthcare needs (16).

#### Healthcare workforce strain

Healthcare workers, including doctors, nurses, and support staff, faced immense pressure and fatigue due to long working hours, high patient loads, and exposure to the virus. Additionally, there were reports of healthcare worker shortages in some areas (19).

#### Infrastructure expansion efforts

To meet the increased demand, India initiated efforts to rapidly expand healthcare infrastructure. This included converting large facilities like stadiums and hotels into makeshift COVID-19 treatment centers, and setting up temporary hospitals in various regions (20).

#### Challenges in rural healthcare

Rural areas faced unique challenges, including limited access to healthcare facilities, shortages of medical supplies, and a lack of healthcare personnel. This highlighted existing disparities in healthcare between urban and rural areas (21).

#### Vaccination campaign and logistics

India launched one of the largest vaccination campaigns in the world, aiming to vaccinate a significant portion of its population. This effort required extensive logistical planning and resources to ensure the efficient distribution of vaccines.

#### Economic impact on healthcare

The pandemic had economic repercussions that affected the healthcare sector. Private healthcare providers faced financial challenges due to reduced non-COVID patient flow and increased costs related to COVID-19 preparedness and treatment (22).

#### Digital health adoption

The pandemic accelerated the adoption of digital health technologies in India. Telemedicine and virtual consultations became crucial tools to provide healthcare services while minimizing in-person contact (23).

#### Mental health strain

The pandemic also had a significant impact on mental health. The stress and uncertainty caused by the pandemic, along with isolation measures, led to increased demand for mental health services (24).

#### Other healthcare services

Routine immunization programs, treatment of communicable and non-communicable diseases experienced interruptions potentially impacting the health and wellbeing of individuals with preexisting conditions (25). Non-emergency medical procedures were postponed, outpatient clinics faced limitations in their operation to accommodate the surge in COVID-19 cases. Elective surgeries were also deferred to prioritize the immediate and critical healthcare needs of COVID-19 patients. These effects have created a wide gap in the provision of healthcare services in managing chronic cases (26).

The pandemic also illuminated preexisting vulnerabilities within India’s healthcare infrastructure, particularly in rural and underserved areas. Limited access to quality healthcare facilities, inadequate staffing levels, and gaps in essential medical supplies became more apparent as the healthcare system grappled with the influx of COVID-19 cases. Similarly, vulnerable communities, such as low income individuals, migrant workers, people in remote areas often faced challenges in obtaining necessary care and medications, further amplifying health disparities (27). Socio-economic disparities, lack of access and inadequate coverage of health care services have left these communities at a higher risk. The economic impact of the pandemic, with job losses and income disparities, exacerbated access issues to healthcare to older adult (28). Furthermore, the COVID-19 pandemic has introduced new challenges, potentially impacting the progress made in the fight against these NTDs.

### Direct impact of COVID-19 on neglected tropical diseases

The COVID-19 pandemic had a direct impact on the prevention, control, and management of Neglected Tropical Diseases (NTDs) in India. NTD programs faced disruptions due to the redirection of resources and attention towards the COVID-19 response. Here are some keyways in which NTD programs were affected:

#### Resource diversion

Resources that were originally allocated for NTD control programs were redirected towards COVID-19 response efforts. This included funding, personnel, and medical supplies that were needed to combat NTDs (29).

#### Disruptions in mass drug administration (MDA)

MDA campaigns, which are crucial for treating and preventing NTDs like lymphatic filariasis and soil-transmitted helminthiasis, faced interruptions. Lockdowns, restrictions on movement, and prioritization of COVID-19 services led to challenges in implementing MDA programs (30).

#### Limited access to healthcare services

Restrictions on movement and healthcare facility capacities impacted the accessibility of services for NTD patients. Individuals may have faced difficulties in seeking diagnosis, treatment, and follow-up care for NTDs.

#### Impact on surveillance and data collection

Data collection and surveillance efforts for NTDs were affected. Field activities, such as surveys and assessments, were hampered due to safety concerns and logistical challenges (29).

#### Disrupted field activities

Lockdowns, travel restrictions, and safety concerns hindered fieldwork for NTD surveillance. This affected data collection and reporting on disease prevalence and distribution.

#### Reduced access to healthcare facilities

Movement restrictions and fear of exposure to COVID-19 led to a decrease in NTD patients seeking diagnosis and care at healthcare facilities (31).

#### Delayed diagnostic testing

Non-essential medical procedures were postponed, potentially leading to delays in diagnostic testing for NTDs.

#### Treatment

Interrupted Mass Drug Administration (MDA) campaigns, critical for diseases like lymphatic filariasis and soil-transmitted helminthiasis, faced interruptions due to restrictions on gatherings and community mobilization efforts 32.

#### Disrupted vector control efforts

Programs aimed at controlling disease-carrying vectors, such as mosquitoes, may have been hampered by lockdowns and resource reallocation.

#### Delayed diagnosis and treatment

The focus on COVID-19 led to delays in the diagnosis and treatment of NTDs. This could potentially lead to an exacerbation of existing NTD cases and hinder progress towards elimination goals (33).

### Indirect impact of COVID-19 on neglected tropical diseases

The burden of Neglected Tropical Diseases (NTDs) in India may have increased because of the indirect effects of the COVID-19 pandemic, such as financial troubles, lockdowns, restricted access to healthcare, and other pathways (34).

#### Economic difficulties

People’s capacity to finance healthcare and NTD prevention measures may have been hampered by economic shocks, job losses, and reduced income levels (35).

#### Limited access to healthcare

Movement restrictions and lockdowns may have made it more difficult for people to get the NTDs they needed to be diagnosed, treated, and followed up on.

Furthermore, it is possible that limits on gatherings and travel hampered NTD control initiatives (34).

#### Diverted resources

It is possible that a reduction in the provision of services for NTDs, such as diagnosis, treatment, and prevention, was caused by the allocation of healthcare resources to COVID-19 response efforts.

#### Disruptions in vector control

Efforts to manage disease-carrying vectors may have been hindered by lockdowns and limits on public health activities, which may have exacerbated the spread of vector-borne NTDs (35).

#### Disrupted outreach and education

Outreach and education initiatives may have been hindered by limits on meetings and community mobilization activities, which may have had an impact on community engagement and awareness- raising activities linked to NTDs (34, 35).

#### Impact on mass drug administration (MDA) programs

MDA programs for NTDs frequently call for extensive drug distribution to vulnerable groups. These programs were interfered with by the COVID-19 pandemic, which delayed the timely administration of medications for NTDs such as lymphatic filariasis and onchocerciasis (34).

#### Delayed surveillance and reporting

Delays in the surveillance and reporting of NTD cases may have occurred due to the focus on COVID-19 surveillance and response. This lag could make it more difficult to identify and contain NTD outbreaks early, possibly allowing them to spread (35).

#### Challenges in conducting research

Due to resource allocation issues and limitations on research efforts, NTD research and clinical trials may have been halted or postponed. This might have an impact on the creation of novel NTD therapies and interventions (34, 35).

#### Impact on vulnerable populations

Both COVID-19 and NTDs may have had a disproportionately negative impact on vulnerable people, such as those who are poor or reside in rural locations. The danger of NTD transmission is increased in these populations by the lack of access to clean water, sanitary facilities, and healthcare (35).

#### Need for integration

Integration is becoming more and more necessary to coordinate NTD control initiatives with larger health systems and pandemic preparedness. Effectively addressing NTDs can be improved by strengthening health systems to respond to COVID-19 (34).

#### Adaptation and resilience

Despite the difficulties the pandemic has presented, there have been instances of adaptability and resiliency in NTD control efforts. Innovative methods have been investigated to lessen the effect, including the use of mobile health devices, community-based interventions, and telemedicine for diagnosis and treatment (34, 35).

### Response and adaptation strategies

The COVID-19 pandemic exacerbated the strain on healthcare resources in countries like India, leaving neglected diseases even more neglected (36). Activities such as mass drug administration, case detection, treatment, and vector control were severely disrupted. Furthermore, access to healthcare systems for patients with NTDs was compromised due to the overwhelming demand and limited availability of beds and timely attention. Issues such as fragmentation within modern healthcare services have resulted in duplication, inefficiencies, subpar outcomes, and an unsatisfactory care experience (29).

This disruption has resulted in a delay or even suspension of crucial NTD prevention and control activities, such as large-scale preventative chemotherapy campaigns. As highlighted in the WHO pulse survey, 44% of countries reported disruptions to NTD services, with 60% reporting disruptions to such campaigns. The World Health Organization developed technical guidance on the implementation of NTD activities in the context of the COVID-19 pandemic guidelines include recommendations on which activities to prioritize, such as the suspension of most community-based activities while continuing essential health facility services and vector control measures (34).

In response to the impact of COVID-19 on NTDs in India, various adaptive measures have been taken to address the challenges and mitigate the effects of the pandemic. These measures include integrating services for COVID-19 and NTDs, promoting interdisciplinary research and collaboration, and ensuring the availability of necessary resources for disease control and elimination efforts. Over the years, it has been highlighted (37) that integrating healthcare services across various sectors has the potential to be advantageous, particularly in resource-constrained settings. Moreover, the capacity to absorb and adjust to disruption is enhanced by the integration of services within the health system and across other sectors. In new roadmap for NTD 2021–2030, emphasis was given on integration of services (35).

**Figure 1:**
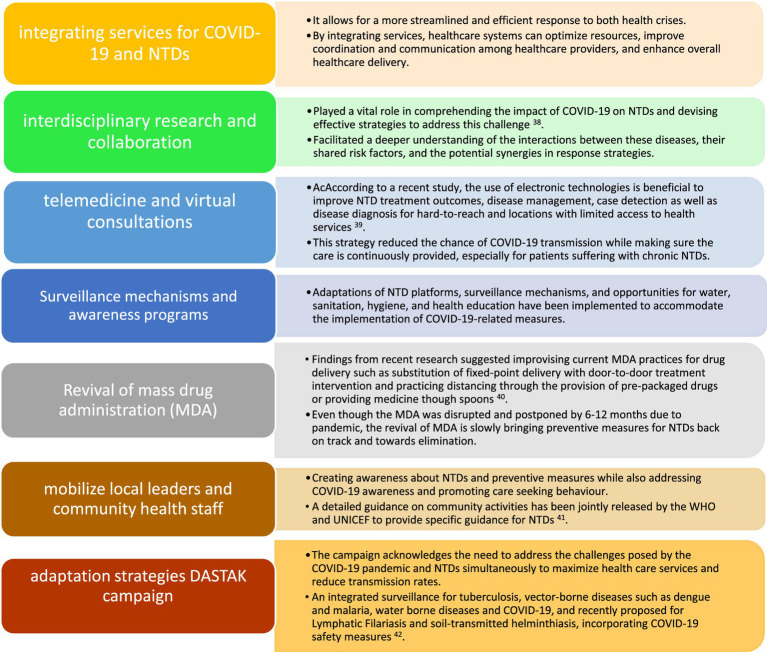
Response and adaptation strategies to address the impact of COVID-19 on neglected tropical diseases in India.

Integrating services for COVID-19 and NTDs has proven to be an effective approach in managing the dual burden of these diseases (Figure 1). The healthcare system collaborated with public health bodies to adapt various measures to integrate services that address both COVID-19 and NTDs, to tackle the challenges posed by the COVID-19 pandemic and mitigate its Impact on NTDs. Moreover, telemedicine and virtual consultations has been recognized as an effective strategy for managing NTDs during the COVID-19 pandemic in India.

These adaptable actions showed how the Indian healthcare system was able to cope with the combined threats posed by COVID-19 and NTDs. However, depending on variables including the local environment, the incidence of diseases, and the state of the healthcare system, these strategies efficacy may differ. It’s crucial to keep tracking and assessing how these actions affects NTD prevention efforts.

### Lessons learned and recommendations

The Coronavirus Disease 2019 (COVID-19) pandemic has highlighted the complex interplay between infectious diseases, including Neglected Tropical Diseases (NTDs), and global healthcare systems. As healthcare systems worldwide grappled with the challenges posed by COVID-19, the noticeable impact on NTD control efforts in low- and middle-income countries (LMICs), such as India became increasingly evident, primarily due to the challenges faced by the healthcare services. This article explores the lessons learned from assessing the consequences of the COVID-19 pandemic on NTDs in India, shedding light on several vital components which are needed to be addressed to improve the healthcare system and the interconnectedness of health crises, resource allocation challenges, and the vital role of community engagement. Furthermore, it offers recommendations to better integrate and fortify NTD programs within pandemic preparedness and response strategies.

### Emergency preparedness

Overwhelmed health systems and limited access to care result in significantly higher mortality rates, both directly from the outbreak and indirectly from preventable conditions. To maintain public trust and ensure appropriate care-seeking behavior, health systems must demonstrate their capacity to provide essential care safely while effectively controlling infection risks. The ability to maintain essential health services depends on disease burden, transmission levels, and health system capacity. Investments in strengthening primary healthcare and implementing strategic adaptations are crucial to address the evolving pandemic context. Safe patient flow, governance, coordination, and adaptation protocols are essential to prevent system failure. As the pandemic evolves, adaptations should be dynamic, and decisions should align with national policies, engaging communities, and stakeholders transparently. Throughout this process, ethical principles and rigorous infection control measures should be the cornerstones of decision-making, with a particular focus on safeguarding vulnerable populations. This article aims to support decision-makers and healthcare managers at both national and subnational levels, ensuring the continuity of essential health services during pandemics (38).

### Robust health infrastructure and capacity building

Enhancing passive screening capacity through the utilization of Rapid Diagnostic Tests (RDTs) and integrating NTD management into routine healthcare services through workforce training is a vital strategy. This approach not only enables the early detection and treatment of NTDs but also mitigates the risks typically associated with large-scale Mass Drug Administration (MDA) programs. By strengthening the skills of healthcare workers and ensuring the safety of both patients and staff, we can effectively manage NTDs, safeguard healthcare infrastructure, and make a significant contribution to the overall public health response during these challenging times (39).

### Robust surveillance and data management

To effectively address the intersection of COVID-19 and NTDs, a prioritized approach to data collection through the health management information system is crucial. This effort should encompass the collection of basic minimum data, including the number of individuals tested, number of positive cases (specifying parasite species and severity), age, and COVID-19 status, where available. Whenever feasible, conducting simultaneous testing for both malaria and COVID-19 is recommended for a more comprehensive understanding of the health landscape. While collecting comprehensive data remains the long-term goal, streamlining notification and investigation processes to minimize surveillance officers’ exposure to the risk of transmission is necessary. Simplifying investigations to primarily focus on vector control coverage, with deficiencies addressed via telephone where possible. It’s important to resume regular notification and investigation processes once transmission has been effectively interrupted, ensuring the continuity of NTD control efforts (38).

### Prioritize essential medicines and supplies

Developing effective strategies to readily identify regions with high endemic disease burdens and ensuring a continuous, dependable drug supply for treatment is essential for addressing the needs of vulnerable populations at greater risk. This necessitates meticulous planning for both local and regional distribution and transportation systems. Furthermore, it involves not only the provision of medicines but also diagnostics and equipment for the management of various high-risk diseases. The establishment of strategic stockpiles and strengthening supply chain resilience play vital roles in achieving these objectives (40).

### Mass drug administration (MDA)

Conducting MDA campaigns for NTDs amid the backdrop of the COVID-19 pandemic presents a series of challenges such as ensuring the safety of healthcare workers and recipients, modifying delivery mechanisms to reduce crowds, emphasizing community engagement and education on both NTD control and COVID-19 prevention, and considering local COVID-19 prevalence to adjust campaign strategies. Striking a delicate balance between the provision of essential health services and rigorous virus prevention through flexibility and adaptability is crucial in this endeavors (41).

### Digital health and telemedicine

In the management of NTDs amid the challenges posed by the COVID-19 pandemic, it has become evident that leveraging digital health technologies, including telemedicine, telehealth, and SMS-based solutions, can provide versatile tools for remote healthcare services, such as NTD screening and patient monitoring. Nevertheless, various challenges such as limited healthcare infrastructure, financial constraints, and regulatory issues, need to be addressed. To navigate these challenges successfully, we recommend expanding community networks, particularly in underserved areas, revising policies related to mobile health (mHealth) concerning NTD management, and formulating sustainable strategies to promote the widespread adoption of digital health technologies in NTD control efforts during the pandemic-like situations. This approach ensures the maintenance of essential healthcare services for NTDs while considering the unique challenges posed by the pandemic (42).

### Community engagement, education, and a multi-sectoral approach

These critical components form a crucial strategy to tackle the challenges posed by emerging and re-emerging diseases, including zoonoses, in the future. The limitations exposed by many countries in responding to the COVID-19 pandemic underscore the urgent need for substantial changes in our healthcare systems and disease surveillance. To better prepare for such threats, it is imperative to foster active community involvement, raise public awareness through education, and implement comprehensive strategies that involve multiple sectors. These changes are essential to enhance our capacity to effectively manage and respond to emerging diseases and zoonotic outbreaks in the future (43).

### Advocacy for resource allocation

Advocating for resource allocation and sustainable funding is crucial, particularly in pandemic situations where NTDs disproportionately affect the most socially and economically vulnerable individuals in LMICs. These populations already face significant barriers to accessing healthcare due to reduced healthcare accessibility and availability. The solution lies in securing robust, predictable, and continuous international and domestic financing, along with political commitment and fostering regional collaboration at the highest levels. Central governments should optimize their roles as control systems to maintain standardized services and monitor local health authorities, providing guidelines for NTD program implementation during the COVID-19 pandemic. Recognizing the importance of leadership and central government control in addressing leprosy program challenges during this crisis, there’s a pressing need to advocate for early detection to strengthen the health system, backed by sustainable funding from both state and central government (44). Implementing these recommendations will contribute to building a more resilient healthcare system in India, capable of effectively responding to future crises while maintaining the integrity of essential health programs.

## Strengths and limitations

This study’s extensive literature evaluation, which uses a scoping review technique to methodically explore the body of knowledge on the effect of the COVID-19 pandemic on neglected tropical diseases (NTDs) in India, is one of its main strengths. Furthermore, the study sheds light on a significant but little-studied topic by concentrating exclusively on NTDs in the context of the COVID-19 pandemic, which may help guide future research and policy decisions. The study’s timeliness is noteworthy as well, since its insights can influence how pandemics are responded to now and in the future, especially when it comes to NTDs. In addition, the research demonstrates adaptive strategies that were implemented to reduce the pandemic’s effects, making it a crucial instrument for legislators and medical professionals.

However, the study has a number of limitations. A primary limitation is the lack of quantitative evidence to substantiate the conclusions. The majority of the qualitative insights offered by the examined studies are valuable, but they cannot be precisely measured to determine the impact. The inability to quantify the effects covered in the review may result from this lack of quantitative data.

Because the study is based on published literature, it may also be impacted by publication bias. Research with noteworthy outcomes are more likely to be published, but underrepresentation of studies with null or negative results might affect the review’s conclusions. Although the regional focus on India adds depth, it may also limit the significance of the results to other areas with distinct healthcare systems and socioeconomic backgrounds. India’s particular problems and solutions might be different from those in other NTD-affected nations.

## Conclusion

In conclusion, even though our analysis emphasizes the serious obstacles the COVID-19 pandemic presents for neglected tropical diseases (NTDs) in India, it’s critical to identify areas in which progress can be made. Although adaptation strategies have been implemented to mitigate the pandemic’s influence on non-transgenic diseases (NTDs), critical analysis is necessary due to underlying constraints and weaknesses. The successful implementation of the adopted adaptive methods is one important factor that needs close examination. Although the integration of COVID-19 and NTD services exhibits possibilities, a thorough assessment is necessary to determine their practical effects on disease prevention and control. To guarantee long-term efficacy, these interventions’ scalability and sustainability also need to be carefully evaluated.

More focus should be given to the socioeconomic factors that influence the prevalence and transmission rates of NTDs. Tackle the underlying causes of NTDs and create resilient healthcare systems by addressing systemic injustices, poverty, and poor sanitary conditions. Acknowledging the ongoing problems and complications inherent in handling the COVID-19 pandemic and NTDs simultaneously is crucial to adopting a more realistic perspective. Through a critical assessment of existing approaches and the identification of opportunities for enhancement, we can enhance our readiness for upcoming health emergencies and protect the welfare of marginalized communities in India and abroad.
